# Designing and Implementing an Implantable Wireless Micromanometer System for Real-Time Bladder Pressure Monitoring: A Preliminary Study

**DOI:** 10.3390/s20164610

**Published:** 2020-08-17

**Authors:** Yu-Ting Li, Ling-Yu Yang, Wei-Ting Hsu, Chih-Wei Peng

**Affiliations:** 1 Taiwan Instrument Research Institute, National Applied Research Laboratories, Hsinchu 30261, Taiwan; ytl@tiri.narl.org.tw (Y.-T.L.); 1709867@tiri.narl.org.tw (W.-T.H.); 2 School of Biomedical Engineering, College of Biomedical Engineering, Taipei Medical University, Taipei 11031, Taiwan; allison73323@gmail.com; 3 International PhD Program in Biomedical Engineering, College of Biomedical Engineering, Taipei Medical University, Taipei 11031, Taiwan

**Keywords:** implantable biomicrosystem, bladder pressure, wireless, biocompatibility

## Abstract

Many mini-implantable devices have been developed and fabricated for diagnostic and treatment purposes. Wireless implantable biomicrosystems provide a desirable approach for long-term physiological signal monitoring. In this study, we implemented a wireless implantable biomicrosystem for bladder-cavity pressure measurements in a freely moving rabbit. To manage the power more effectively, a magnetic reed switch was applied to turn on/off the implantable module using a neodymium–iron–boron (NdFeB) magnet. The measured bladder pressure signal was wirelessly transmitted from the implantable module to a host unit. Our results indicated that the implantable biomicrosystem exhibited satisfactory performance and safety, as evidenced by an error percentage of less than ±1% for pressure measurements and less than 2 °C of a temperature rise under normal operation. The wireless biomicrosystem was implanted into the bladder cavity of a rabbit. Bladder pressure was simultaneously measured by both the biomicrosystem and conventional cystometry in the animal. The two signals were similar during the voiding phase, with a correlation coefficient of 0.885. Additionally, the biomicrosystem coated with polydimethylsiloxane in this study showed no cytotoxicity, which confirmed its biocompatibility. In conclusion, we demonstrated a good biocompatible wireless biomicrosystem which showed good reproducibility with respect to pressure monitoring by conventional cystometry. Further studies are needed to confirm the results of this preliminary feasibility study for actual clinical applications.

## 1. Introduction

The lower urinary tract (LUT) comprises the reservoir (urinary bladder), and the outlet (bladder neck, urethra, and urethral sphincter) is used for the storage and periodic elimination of urine. Normally, the two main structures exhibit reciprocal activity. During urine storage, the urinary bladder is quiescent and the activity of the outlet gradually increases; in contrast, the bladder is contracted and the outlet is relaxed when urine is emptied [1]. However, various physiologic and anatomical deficits can cause disorders of LUT functions, such as incontinence and urine obstruction. Such LUT disorders can cause detrusor hypertrophy/hyperplasia, overactive bladder, high voiding pressure, vesicoureteral reflux, and even death due to complications [2,3,4,5]. Many of these bladder disorders can be predicted and prevented by monitoring variations in abnormal bladder pressure. Thus, bladder pressure measurements provide critical information for evaluating the state of bladder conditions in patients suffering from LUT disorders.

In clinical urodynamic evaluations, bladder assessments are usually performed in the hospital by inserting a very long catheter into the bladder cavity for filling and emptying the bladder [6,7]. The catheter passes through the urethra and connects to external equipment, which can cause infections during long-term monitoring, and it is uncomfortable, painful, and non-physiological for patients. Therefore, developing a small, light, wireless mini-implantable biomicrosystem is essential for chronic and long-term bladder pressure monitoring [8,9]. In recent years, increasing numbers of wireless mini-implantable systems have been developed for bladder pressure and volume measurement experiments; they were designed to be placed into the bladder cavity or on the bladder wall and wirelessly transmit measured data [10,11,12,13,14,15,16,17,18]. A battery-powered telemetric pressure-sensing system with a pressure sensor die packaged in a small catheter was developed using a wireless transmitter module [14]. Although it was successfully implemented in animal studies lasting over 3 days, the measurement module was too large to be fully implanted into the bladder cavity. Another telemetric-pressure monitoring system with wireless power was designed using Class-E amplifiers to provide power to the internal module which allowed it to be used for long-time implantation studies [11]. However, the transmission distance of the magnetic coupling technique is usually less then several centimeters which might restrict the movement of patients. An improved telemetric pressure-measurement system with wireless battery charging was also developed [12,16,17].

To date, few small light wireless bladder pressure-sensing systems have been developed and successfully applied for in vivo animal experiments by fully implanting the module into the bladder cavity [14,18]. Those studies would be commonly characterized with high charging module temperature, short transmission distance, and insufficient power energy or loss data while the external and internal coils are inappropriately arranged in horizontal direction. Compared to those studies, the present study implemented a fully implantable, safe (low temperature rise), long transmission distance, and non-directional wireless biomicrosystem that can provide bladder pressure measurements for evaluating the state of bladder conditions in animal experiments. With our wireless transmission scheme, the problem of catheters causing urethral infections and movement restrictions during long-term monitoring can be avoided. The battery powering the implantable device was also managed to extend its life for long-term monitoring purposes. Finally, the entire module was hermetically encapsulated to protect the implantable device and coated with a silicone elastomer for biocompatibility, and then applied in in vivo animal experiments.

## 2. Materials and Methods

### 2.1. Wireless Implantable Biomicrosystem

**Figure 1:**
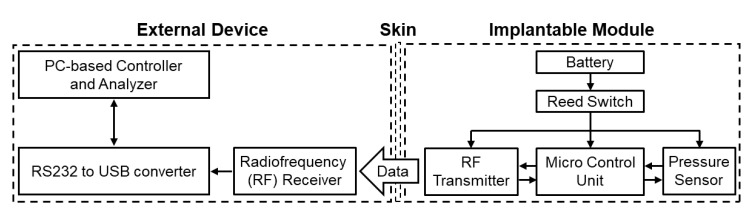
System block diagrams of the wireless implantable biomicrosystem for bladder pressure monitoring. RF is defined as radiofrequency.

Block diagrams of the wireless implantable biomicrosystem for monitoring bladder pressure are shown in Figure 1. The entire wireless system consists of a host unit for collecting data and an implantable unit for detecting bladder pressure. The host unit includes a radiofrequency (RF) receiver, an RS232-to-USB converter, and a personal computer (PC). The implantable unit contains a precision pressure-sensing module, a microcontroller unit (MCU), an RF transmitter, a reed switch, and a battery.

**Figure 2:**
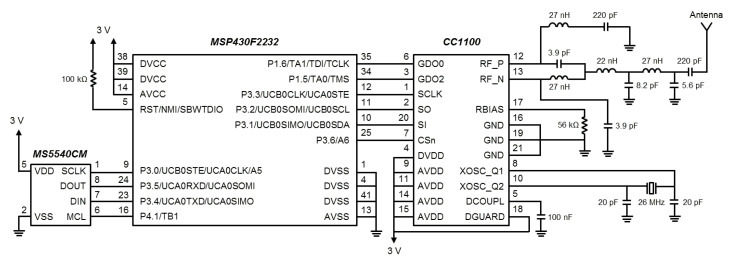
Overall schematic diagram of the wireless pressure measurement biomicrosystem.

In this system, pressure was detected using a miniature pressure sensor (MS5540CM, Intersema) which was comprised of a precision piezoresistive pressure sensor and 16-bit analog-to-digital converter (ADC). Ring-shaped special anticorrosive and antimagnetic stainless steel were used to encircle and protect the pressure-sensor chip and ADC. This hybrid pressure sensor requires no external components such as an amplifier circuit or baseline compensation circuit, which facilitate miniaturizing the system size and reducing power consumption. The pressure sensor was controlled by the serial peripheral interface (SPI) of the MCU (MSP430F2232) to read digital pressure values. After the MCU detects the digital pressure value from the pressure sensor, the data were encoded into a universal asynchronous receiver/transmitter (USART) form and wirelessly transmitted at a carrier frequency of 433 MHz via a miniature RF transmitter (CC1100) to the host unit. Finally, pressure signals were displayed in real time on a graphical user interface (GUI) written in LabVIEW and stored in the PC. The detailed circuit of the wireless bladder pressure measurement system is shown in Figure 2.

The entire implantable biomicrosystem is powered by a 3-V lithium–manganese dioxide battery (EEMS, CR 1/3N, 160 milliamp-hours (mAh)) which has a low self-discharge of less than 1% per year. To manage the power more effectively, several methods were used to reduce the power consumption and extend the battery life. Since it might be unnecessary to continuously monitor the bladder pressure all day long in real clinical applications, a neodymium–iron–boron (NdFeB, dimension: 5 (L) × 5 (W) × 5 cm (H), weighing 900 g) magnet was used to turn on/off the momentary type of reed switch (ORD 211), which commonly has a life expectancy of more than 5 × 10^6^ switching cycles (testing at 12V_dc_ and 100 mA) and a pull-in value between 10 to 40 AT. The maximum strength of the magnetic field is 12.1, 4.7, and 3.0 mT away from the NdFeB magnet surface of 10, 15, and 18 cm, respectively. Thus, the effective control distance between the magnet and reed switch can reach at least 18 cm. The reed switch is magnetically switched on to activate all electrical components and detect the pressure value at a sampling rate of 5 Hz when the magnet is placed close to the implantable module, whereas the system is switched off when the magnet is further away from the module. Second, the entire implantable module is set in sleep mode except for the detection and transmission phases. Finally, the sampled pressure data are stored in the memory of the MCU and transmitted every five measurements.

After assembling the entire implantable biomicrosystem, it was coated with several thin layers of epoxy and polydimethylsiloxane (PDMS) to provide watertight protection. For system encapsulation, an epoxy adhesive (ECP-1330; Slink; part A: part B = 1:1) was applied several times around the electronic components on the printed circuit board (PCB) except for the pressure sensor until all the components and edges of the PCB were fully covered, and each application step was allowed to cure for 24 h. Finally, the entire system was immersed in a PDMS solution (Sylgard 184; Dow Corning; silicone elastomer: curing agent = 10: 1) to ensure biocompatibility [19].

### 2.2. System Validation

A range of 0~250 cmH_2_O and pressure resolution of 1 cmH_2_O are recommended for bladder pressure monitoring [20]. To validate the telemetric pressure module, the sealed implantable device was placed at the center of a hermetically sealed container (8 (Ø) × 10 cm (H)) filled with a 0.9% saline solution to verify its water-tightness and wireless pressure measurement. A commercial precision pressure calibrator (718-30G, Fluke) with a pressure hand pump was linked to the container to control and monitor the inner pressure values which were set to 0~250 cmH_2_O in 50 cmH_2_O increments in the validation experiment. After the absolute pressure was measured by the hybrid pressure sensor, the atmospheric pressure on the test day and hydraulic pressure of the saline solution were subtracted from the measured pressure value to obtain the container’s inner pressure. Validation experiments were performed on days 0, 3, 7, 14, and 21 after the implantable module was placed into the saline solution. The inner pressure of the hermetically sealed container was kept at 100 ± 10 cmH_2_O for 3 weeks except for the validation period. A pressure of 100 cmH_2_O was chosen in this study because this is the maximum value of bladder pressure in animal and clinical experiments. Finally, the results measured by our wireless pressure system were compared to those obtained using a commercial precision pressure meter. The output error percentage of the pressure at various loads was calculated. The error percentage of the pressure output was calculated as follows:

(1) $$\begin{array}{c} Pressure\;Error\;Percentage\;\left(\%\right)\\ \;=\frac{Expected\;pressure\;value-\;Measured\;pressure\;value}{Measured\;pressure\;value} \end{array}$$

### 2.3. Safety Validation

To ensure safety, international standard ISO 14708-1 specifies that the outer surface of an implantable part of an active implantable medical device shall not exceed 39 °C in a test ambient temperature of 37 °C. Before the temperature test, heat sources of our implantable module, including the MCU and crystal, were detected using an infrared thermal imaging camera (E6, FLIR, Wilsonville, OR, USA). Then T-type thermocouple wires were glued to the outer surface of the module which is close to the MCU and crystal using cyanoacrylate adhesive (D-3 and 606, Satlon, Taiwan). The entire module was then placed into a temperature and humidity chamber (THS-D4T-100, KSON, Taiwan), and the temperature of the module under normal operation was recorded in an ambient test temperature of 37 °C using a data logger (GL820, Graphtec, Yokohama, Japan).

### 2.4. Animal Experiment

The implantable biomicrosystem was covered with PDMS for in vivo experimentation. A sterilized biomicrosystem was implanted into the bladder cavity of a male New Zealand white rabbit which weighed 2.0~3.0 kg. The biomicrosystem position was confirmed by x-ray. The rabbit was housed under a 12 h light–dark cycle with ad libitum access to food and water. All experimental procedures used in this study complied with the Institutional Animal Care and Use Committee (IACUC) of Taipei Medical University (IACUC approval no.LAC-2019-0217).

For comparison between our wireless biomicrosystem and a standard clinical diagnostic procedure, we compared the bladder pressures measured by this device and cystometric measurements with a commercial biological signal acquisition system (Biopac MP 36, BIOPAC Systems, Santa Barbara, CA, USA). Cystometry was performed the same way as in our previous studies [21,22,23]. The rabbit was anesthetized with isoflurane (3~5%) through a facemask. After sterilization, the bladder of the rabbit was surgically exposed, and the wireless biomicrosystem was inserted beneath the bladder mucosa to facilitate the fluid pressure measurements. After surgical implantation, the rabbit was returned to its cage and given 1 week to recover from surgery. The rabbit was properly treated to prevent wound inflammation during recovery from the surgery. Bladder pressure was measured 1 week after implantation. To measure cystometry using Biopac MP 36, the rabbit was anesthetized, and a polyethylene tube (PE-50) was inserted into the bladder via the lumen and attached to both a pressure transducer and a syringe pump for filling saline. After closing the bladder incision with a pursestring suture around the PE-50 tube, cystometry was validated by filling the bladder with physiological saline (4 mL/min, at room temperature) which led to micturition [24]. Wireless data and the cystometric signal were simultaneously recorded for 30 min for the correlation analysis. After the recording was completed, the rabbit was sacrificed, and the bladder tissues were examined for the biocompatibility test of PDMS used in the biomicrosystem.

### 2.5. In Vivo Cytotoxicity Tests

Bladder tissues attached to the biomicrosystem coated with PDMS were collected, fixed in 10% neutral formalin for 24 h, and then embedded in paraffin. Serial 4 μm-thick sections were cut and dried overnight at 37 °C. Sections were routinely stained with hematoxylin and eosin (H&E) for pathological diagnosis, as well as Masson’s trichrome stain to identify fibrosis in the mucosa layer. All immunohistochemical (IHC) slides were subsequently examined using light microscopy by a pathologist blinded to the specimen origins.

## 3. Results

### 3.1. Overall Structure of The Wireless Mini-Invasive Biomicrosystem

**Figure 3:**
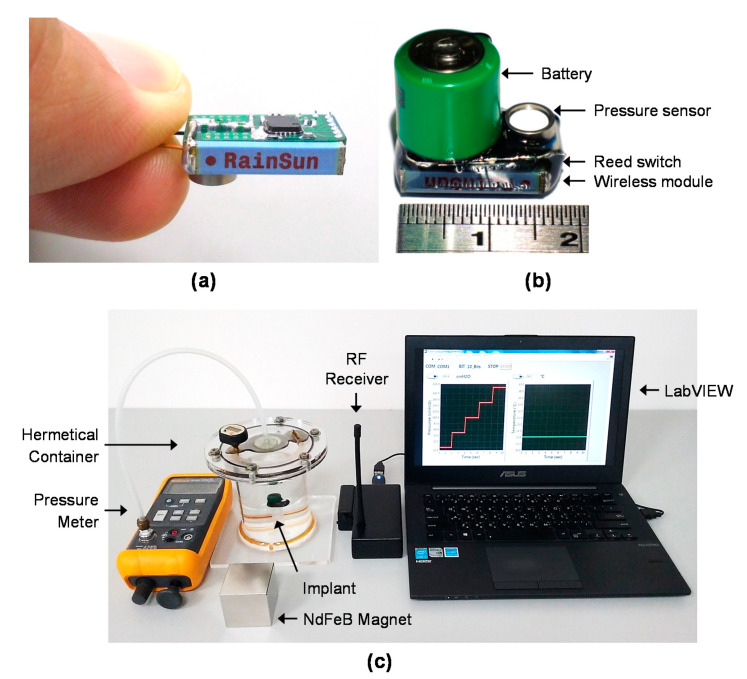
Photos (**a**) before and (**b**) after the hermetic packaging of our implantable pressure sensing system. (**c**) Experimental setup of our system for pressure validation.

The wireless mini-implantable module was fabricated with small components and built on a PCB producing a module with dimensions of 1.9 (L) × 1.2 (W) × 1.8 cm (H) and a total weight of about 5 g, which is suitable for long-term rabbit bladder implantation. The entire implantable biomicrosystem was covered with epoxy and PDMS for biocompatibility, as depicted in Figure 3a,b. Pressure values were detected at 5 Hz, and these data were transmitted every second at a carrier frequency of 433 MHz which was modulated using minimum shift keying (MSK). Maximum data rates of the RF transceiver (CC1100) could be set up to 500 kbps and with less than a 1% packet error rate. The maximum current consumption of the implantable module was about 25 mA during the data transmission. Our implantable biomicrosystem was able to transmit the data to the RF receiver at up to 2 m away while implanted. Detailed specifications of the implantable module are summarized in Table 1.

### 3.2. Experimental Setup For System Validation

**Figure 4:**
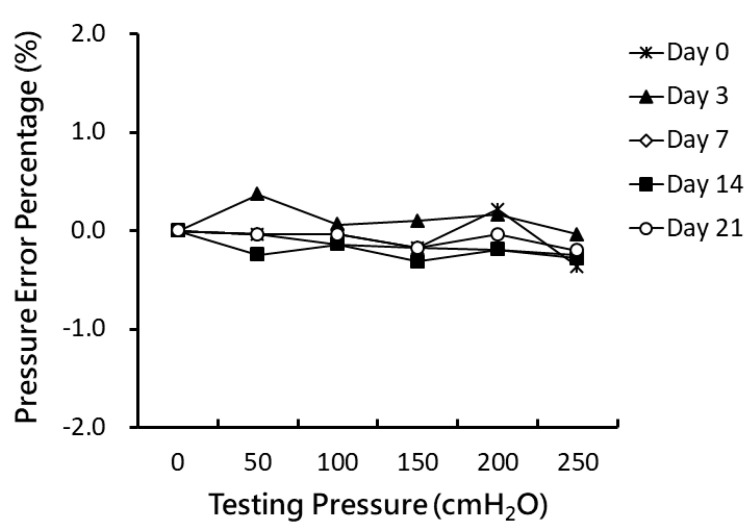
Pressure output errors for various loads of 0~250 cmH_2_O at 50 cmH_2_O increments on days 0, 3, 7, 14, and 21.

Wireless pressure measurements were validated by placing the implantable module into a hermetically sealed container filled with saline solution, as depicted in Figure 3c. The hermetically sealed implantable module could successfully transmit the measured pressure data externally to the host unit. Comparisons of pressure values were obtained using a commercial precision pressure meter and our wireless pressure sensing module on days 0~21. Figure 4 depicts the error percentage of the pressure output as the load was varied from 0 to 250 cmH_2_O in 50 cmH_2_O increments, which was used to simulate various bladder pressures. The error percentages of pressure output were all within ±1% of loads of 0~250 cmH_2_O during the 3 week experimental period.

### 3.3. Safety Validation

**Figure 5:**
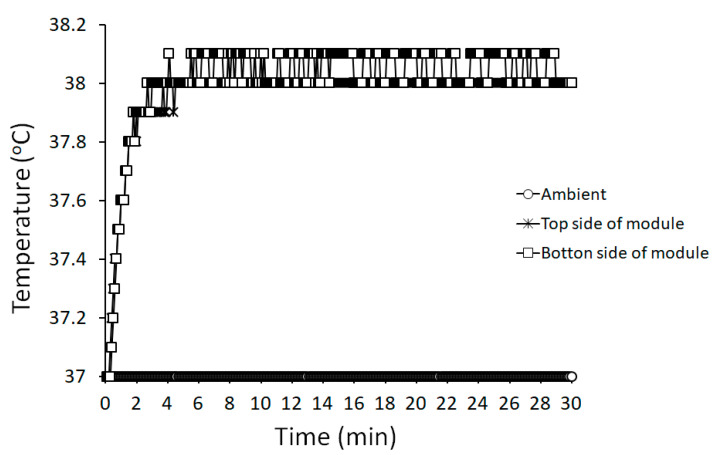
Temperature rise curve of our implantable biomicrosystem under normal operation.

Figure 5 shows the temperature curve of the module under normal operation in the ambient test temperature of 37 °C. The measured temperatures on the upper and lower surfaces of module rose to about 38 °C within 3 min and maintained thermal stability at 38~38.1 °C for more than 27 min. Therefore, the temperature rise test of our implantable module was in compliance with the limitations of international standard ISO 14708-1 when the module was operated under normal conditions.

### 3.4. Animal Experiment

**Figure 6:**
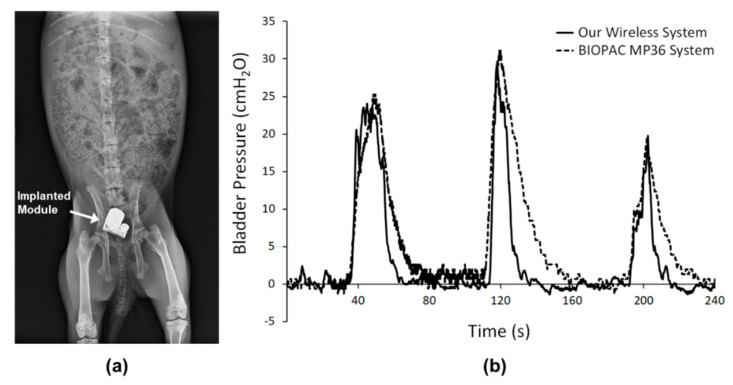
(**a**) X-ray photograph of the biomicrosystem implanted in the bladder cavity of a rabbit. (**b**) Comparison of the inner bladder pressure signals recorded using our wireless system (solid line) and a commercial biological signal acquisition system (dashed line).

In our in vivo experimental measurements, the implantable biomicrosystem covered with PDMS was implanted into the bladder cavity of a rabbit for 1 week. Figure 6a shows an X-ray image of the biomicrosystem implanted into the rabbit’s body. Bladder pressure signals were simultaneously captured using our wireless system and a commercial biological signal acquisition system (Biopac MP 36). Figure 6b shows the bladder pressure versus testing time. Our data showed that the errors of the maximum bladder pressure in each testing cycle were less than 5%, and ranged 95.0~101.5%, as evidenced by the ratio of recorded pressure between our system and Biopac MP 36. The two signals were similar during the voiding phase, but deviations in pressure values during the voiding phase were probably due to the length of the PE tube of the MP 36 sensor during cystometric measurements, which caused the pressure to decrease with a small lag time. The correlation coefficient was 0.885.

### 3.5. In Vivo Cytotoxicity

**Figure 7:**
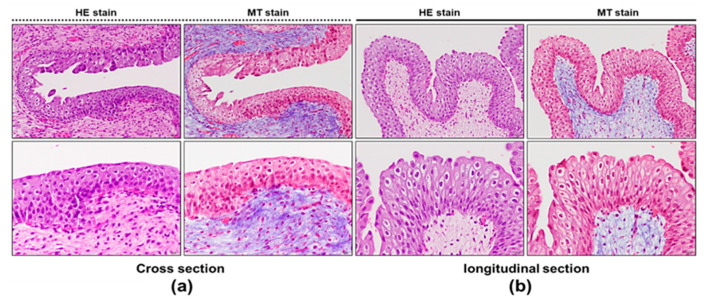
Histological findings of the rabbit bladder at 7 days after implantation. (**a**) Cross and (**b**) longitudinal sections of a representative mucosal layer of the bladder stained with hematoxylin and eosin (H&E) and Masson’s trichrome (MT) (magnification, 100× and 200×).

With regard to biocompatibility, we observed that the biomicrosystem coated with PDMS was easily implanted into the bladder cavity. Bladder H&E staining at 1 week after implantation showed no defects in the transitional epithelium of the mucosal layer (Figure 7). In this study, we also verified the presence of increased amounts of collagen fibers by Masson’s trichrome staining (Figure 7). Masson’s trichrome-stained sections revealed no obvious increase in collagen accumulation in the mucosal layer, indicating that the biomicrosystem did not result in fibrosis development during the implantation period.

## 4. Discussion

For in vivo studies, a mini-implantable wireless biomicrosystem is desirable for the long-term assessment of bladder conditions. Several previous studies proposed and implemented implantable bladder pressure devices, but few studies implanted the entire pressure sensor module into the bladder cavity in animal studies [11,14,15,16]. For example, a relevant study by Tan et al. [14] intracorporeally implanted a bladder pressure sensor in a pig for around 3 days, but their main implantable sensor module was placed in a subcutaneous pocket of the abdomen and was connected to one or two pressure catheter leads. The tip of their pressure catheter lead was inserted and sutured into the bladder cavity through an incision in the bladder wall. Their implant design of leaving the pressure catheter permanently passing through the bladder wall incision may have increased the risk of wound-related infections. In the present study, a mini-implantable wireless biomicrosystem was successfully designed and implemented for monitoring bladder pressure by placing the entire module into the bladder cavity of a rabbit. Therefore, our design has the merit of avoiding wound-related infections compared to that of the previous study. Moreover, our pressure-sensing device could wirelessly transmit measured pressure data to the host unit. The applicable transmission distance was determined to be within 2 m, which is adequate for placing a module into the bladder cavity of a freely moving rabbit for many biomedical applications.

For the long-term implantation of biomedical applications, several factors should be considered, including the accuracy and sensitivity of the system measurements, and the safety of the system. First, our preliminary in vivo results demonstrated that the implantable wireless biomicrosystem could correctly detect bladder pressure with an error of <5%. The low error of the implantable biomicrosystem is acceptable for most clinical applications. Second, our data also showed that the implantable wireless biomicrosystem was more sensitive than the Biopac MP 36 sensor in detecting pressure, especially during the voiding phase. Real-time changes in bladder pressure could be correctly detected using the implantable wireless biomicrosystem, because the pressure sensor of our module was placed inside the bladder cavity. In contrast to the implantable module, the Biopac MP 36 exhibited a slower release of the pressure because it monitored pressure via a long, thin PE tube. Third, there was no corrosion or damage to the implantable module after implantation for 7 days. The implantable module still maintained its essential performance, including pressure measurement and wireless transmission, as evidenced by the sensitivity of our pressure sensor not exhibiting significant changes. The error percentages of the pressure output of the implantable wireless biomicrosystem before and after implantation for 7 days were all within ±1% compared to those of Fluke 718-30G. Finally, our implantable module exhibited a low temperature rise of <1.1 °C, which is biocompatible with bladder tissues, and confirms its safety for long-term implantation. Therefore, our implantable wireless biomicrosystem demonstrated low error, high sensitivity, long-term stability, and safety in pressure measurements, and is thus suitable for animal studies and clinical applications.

Although we successfully measured the pressure changes in a hermetically sealed container and in vivo in a rabbit bladder, minimizing the module size, prolonging the lifetime of the implantable device, and increasing the buoyancy of the implantable device are our immediate goals for improving the present wireless system. The antenna size is a critical factor affecting the implantable module size. A low carrier frequency generally has a larger antenna size and a lower data transmission rate. Therefore, after considering tissue absorption, the data transmission rate, and the antenna size, we used a carrier frequency of 433 MHz for the transmission of the bladder pressure signal in our system. The antennas currently used have dimensions of 16.0 (L) × 3.1 (W) × 1.6 mm (H) (RainSun) and 12.3 (L) × 4.0 (W) × 1.6 mm (H) (YAGEO), which are appropriate for module miniaturization in the future. In addition, the pressure sensor, MCU, and RF dying instead of the surface mount device (SMD) packaged components might be able to miniaturize the implantable module. If the dimensions of the bladder pressure monitoring unit could be reduced to 0.4 (Ø) × 2 cm (L) in a cylindrical shape with a weight of approximately 3 g, then it could easily be in vivo placed into the bladder cavity of a pig via the urethral outlet for non-surgical implantation. This would reduce wound-related infection risk factors. On the other hand, for the power control of the implantable module, we used a momentary type of reed switch, which might not be the optimal practical solution for clinic applications. This is because the heavy magnet should be worn on the human trunk during the working time. Thus, the NdFeB magnet could be replaced by using an electromagnet, which is more easily applied in clinical conditions in the future. In addition, providing power using rechargeable batteries is more suitable for long-term in vivo studies compared to primary batteries, which are currently used to power wireless pressure-sensing modules. Thus, the wireless magnetic coupling technique can be applied to wirelessly charge Li-ion batteries. A high-efficiency Class-E power amplifier can provide a 2-MHz electromagnetic wave to transmit power into the implantable module [25]. Regulated DC power is then delivered into the charge-integrated circuit (IC; LTC4065LX) to charge the rechargeable Li-ion battery. We expected that the improved wireless pressure-sensing system can be applied for more than 1 month. Making the implantable device float in urine is also one of our challenges, because such a property of the device can prevent the bladder outlet from being obstructed, and it can also provide orientation for the wireless power charging. Thus, a small latex balloon can be attached to the tip of the implantable device and then inflated using a syringe pump.

Neuromodulatory techniques are commonly proposed to effectively control the storage and evacuation of urine in urological treatments of voiding dysfunction, by applying electrical stimulation to regulate the reflex activity of the bladder [26,27,28,29]. Many studies demonstrated that the modulation of the pudendal nerve reflex can facilitate reflex bladder contractions and improve bladder capacity in animals and humans [30,31,32,33]. Therefore, pudendal nerve modulation could be an alternative for treating patients with various voiding dysfunctions. In the future, the proposed concept of bladder pressure sensing in a wireless system could be integrated with our previously developed microstimulator and used in urodynamic and neuromodulatory applications. The implantable bladder pressure-sensing device can provide pressure feedback to an external receiver, and a microstimulator could be triggered to deliver a stimulation current via a cuff electrode for bladder modulation [34]. In addition, a similar device can be extended as an experimental platform for various neuroscience studies which require long-term pressure monitoring, such as intracranial pressure (ICP) recording in patients with hydrocephalus, intraventricular hemorrhage, traumatic brain injury (TBI), and stroke [35]. With the proposed wireless module, the changes in pressure levels within the ventricles of the brain could be monitored and the catheter-related infection risk factors could also be avoided.

## 5. Conclusions

An implantable wireless micromanometer system for measuring bladder pressure was implemented. A hybrid pressure sensor, which was comprised of a precision piezoresistive pressure sensor and 15-bit ADC, was used to detect pressure levels in the bladder cavity. Bladder pressure data could be measured and wirelessly transmitted via a wireless module. A reed switch and an NdFeB magnet were used to manage the power to extend the battery life. The entire wireless module was hermetically packaged in silicone to make it biocompatible. The proposed wireless system was successfully validated in a saline solution in a hermetically sealed container for 1 week. The proposed wireless voltammetric system can be extended to many novel human and animal studies, such as in ICP monitoring, which require chronic pressure recording in patients.

## Figures and Tables

**Table 1:** Specifications of the implantable wireless micromanometer system.

**Dimensions**	1.9 (L) × 1.2 (W) × 1.8 cm (H)
**Carrier frequency**	433 MHz
**Transmission distance**	2 m (max.)
**Sampling rate**	5 Hz
**Pressure range**	<1~100 cmH_2_O
**Pressure resolution**	0.1 cmH_2_O

## References

[1] Chang H.Y., Peng C.W., Chen J.J.J., Cheng C.L., de Groat W.C. (2004). The time-frequency analysis of the pudendo-to-pudendal nerve and pelvic-to-pudendal nerve reflexes in anesthetized intact rats. J. Med. Biol. Eng..

[2] Abrams P., Cardozo L., Fall M., Griffiths D., Rosier P., Ulmsten U., Kerrebroeck P.V., Victor A., Wein A. (2002). Standardisation sub-committee of the international continence society, the standardisation of terminology of lower urinary tract function: Report from the standardisation sub-committee of the international continence society. Neurourol. Urodyn..

[3] Ghoniem G.M., Regnier C.H., Biancani P., Johnson L., Susset J.G. (1986). Effect of vesical outlet obstruction on detrusor contractility and passive properties in rabbits. J. Urol..

[4] Sillen U. (2008). Bladder dysfunction and vesicoureteral reflux. Adv. Urol..

[5] Takahashi S., Homma Y., Fujishiro T., Hosaka Y., Kitamura T., Kawabe K. (2000). Electromyographic study of the striated urethral sphincter in type 3 stress incontinence: Evidence of myogenic-dominant damages. Urology.

[6] Nosseir M., Hinkel A., Pannek J. (2007). Clinical usefulness of urodynamic assessment for maintenance of bladder function in patients with spinal cord injury. Neurourol. Urodyn..

[7] Wille S., Schumacher P., Paas J., Tenholte D., Eminaga O., Muller U., Muthen N., Mehner J., Cornely O., Engelmann U. (2014). Catheterless long-term ambulatory urodynamic measurement using a novel three-device system. PLoS ONE.

[8] Dakurah M.N., Koo C., Choi W., Joung Y.H. (2015). Implantable bladder sensors: A methodological review. Int. Neurourol. J..

[9] Yu L., Kim B.J., Meng E. (2014). Chronically implanted pressure sensors: Challenges and state of the field. Sensors.

[10] Chen S.C., Hsieh T.H., Fan W.J., Lai C.H., Chen C.L., Wei W.F., Peng C.W. (2015). Design and evaluation of potentiometric principles for bladder volume monitoring: A preliminary study. Sensors.

[11] Coosemans J., Puers R. (2005). An autonomous bladder pressure monitoring system. Sens. Actuators. A Phys..

[12] Majerus S.J., Garverick S.L., Suster M.A., Fletter P.C., Damaser M.S. (2012). Wireless, ultra-low-power implantable sensor for chronic bladder pressure monitoring. ACM J. Emerg. Technol. Comput. Syst..

[13] Melgaard J., Rijkhoff N.J. (2011). Detecting the onset of urinary bladder contractions using an implantable pressure sensor. IEEE Trans. Neural Syst. Rehabil. Eng..

[14] Tan R., McClure T., Lin C.K., Jea D., Dabiri F., Massey T., Sarrafzadeh M., Srivastava M., Montemagno C.D., Schulam P. (2009). Development of a fully implantable wireless pressure monitoring system. Biomed. Microdevices.

[15] Wang C.C., Huang C.C., Liou J.S., Ciou Y.J., Huang I.Y., Li C.P., Lee Y.C., Wu W.J. (2008). A mini-invasive long-term bladder urine pressure measurement ASIC and system. IEEE Trans. Biomed. Circuits Syst..

[16] Majerus S.J., Fletter P.C., Damaser M.S., Garverick S.L. (2010). Low-power wireless micromanometer system for acute and chronic bladder-pressure monitoring. IEEE Trans. Biomed. Eng..

[17] Young D.J., Cong P., Suster M.A., Damaser M. (2015). Implantable wireless battery recharging system for bladder pressure chronic monitoring. Lab Chip.

[18] Jourand P., Puers R. (2010). The bladder pill: An in-body system logging bladder pressure. Sens. Actuators. A Phys..

[19] Kim S.J., Lee D.S., Kim I.G., Sohn D.W., Park J.Y., Choi B.K., Kim S.W. (2012). Evaluation of the biocompatibility of a coating material for an implantable bladder volume sensor. Kaohsiung J. Med. Sci..

[20] Schäfer W., Abrams P., Liao L., Mattiasson A., Pesce F., Spangberg A., Sterling A.M., Zinner N.R., Kerrebroeck P.V. (2002). International Continence Society, Good urodynamic practices: Uroflowmetry, filling cystometry, and pressure-flow studies. Neurourol. Urodyn..

[21] Jen E., Hsieh T.H., Lu T.C., Chen M.C., Lee F.J., Lin C.T., Chen S.C., Chu P.Y., Peng C.W., Lin C.W. (2017). Effects of pulsed-radiofrequency neuromodulation on the rat with overactive bladder. Neurourol. Urodyn..

[22] Praveen Rajneesh C., Lai C.H., Chen S.C., Hsieh T.H., Chin H.Y., Peng C.W. (2019). Improved voiding function by deep brain stimulation in traumatic brain-injured animals with bladder dysfunctions. Int. Urol. Nephrol..

[23] Praveen Rajneesh C., Yang L.Y., Chen S.C., Hsieh T.H., Chin H.Y., Peng C.W. (2019). Cystometric measurements in rats with an experimentally induced traumatic brain injury and voiding dysfunction: A time-course study. Brain Sci..

[24] Dobberfuhl A.D., Spettel S., Schuler C., Levin R.M., Dubin A.H., De E.J. (2015). Noxious electrical stimulation of the pelvic floor and vagina induces transient voiding dysfunction in a rabbit survival model of pelvic floor dystonia. Korean J. Urol..

[25] Liang C.K., Chen J.J., Chung C.L., Cheng C.L., Wang C.C. (2005). An implantable bi-directional wireless transmission system for transcutaneous biological signal recording. Physiol. Meas..

[26] Kerrebroeck E.V.E., Koldewijn E., Wijkstra H., Debruyne F. (1991). Intradural sacral rhizotomies and implantation of an anterior sacral root stimulator in the treatment of neurogenic bladder dysfunction after spinal cord injury. World J. Urol..

[27] McGee M.J., Amundsen C.L., Grill W.M. (2015). Electrical stimulation for the treatment of lower urinary tract dysfunction after spinal cord injury. J. Spinal Cord Med..

[28] Middleton J.W., Keast J.R. (2004). Artificial autonomic reflexes: Using functional electrical stimulation to mimic bladder reflexes after injury or disease. Auton. Neurosci..

[29] Tai C., Chen M., Shen B., Wang J., Liu H., Roppolo J.R., de Groat W.C. (2011). Plasticity of urinary bladder reflexes evoked by stimulation of pudendal afferent nerves after chronic spinal cord injury in cats. Exp. Neurol..

[30] Peters K.M., Feber K.M., Bennett R.C. (2005). Sacral versus pudendal nerve stimulation for voiding dysfunction: A prospective, single-blinded, randomized, crossover trial. Neurourol. Urodyn..

[31] Vodušek D., Plevnik S., Vrtačnik P., Janež J. (1987). Detrusor inhibition on selective pudendal nerve stimulation in the perineum. Neurourol. Urodyn..

[32] Hokanson J.A., Langdale C.L., Sridhar A., Grill W.M. (2018). Stimulation of the sensory pudendal nerve increases bladder capacity in the rat. Am. J. Physiol. Ren. Physiol..

[33] Peng C.W., Chen J.J., Cheng C.L., Grill W.M. (2008). Role of pudendal afferents in voiding efficiency in the rat. Am. J. Physiol. Regul. Integr. Comp. Physiol..

[34] Li Y.T., Peng C.W., Chen L.T., Lin W.S., Chu C.H., Chen J.J. (2013). Application of implantable wireless biomicrosystem for monitoring nerve impedance of rat after sciatic nerve injury. IEEE Trans. Neural Syst. Rehabil. Eng..

[35] Welschehold S., Schmalhausen E., Dodier P., Vulcu S., Oertel J., Wagner W., Tschan C.A. (2012). First clinical results with a new telemetric intracranial pressure-monitoring system. Neurosurg.

